# Nurses' perceptions of online continuing education

**DOI:** 10.1186/1472-6920-11-86

**Published:** 2011-10-20

**Authors:** Selcuk Karaman

**Affiliations:** 1 Open Education Faculty, Ataturk University, 25240 Erzurum, Turkey

## Abstract

**Background:**

There is increasing attention to online learning as a convenient way of getting professional training. The number and popularity of online nursing continuing education programs are increasing rapidly in many countries. Understanding these may contribute to designing these programs to maximize success. Also, knowing the perceptions and preferences in online learning aids development and orientation of online programs. The aims of this study are to show nurses' perceptions of online continuing education and to determine perceptions of various groups; area groups, working companies, frequency of computer usage and age.

**Methods:**

The survey method was used in this quantitative study to reveal perception levels and relationship with related variables. Data were collected through an online instrument from a convenience sample of 1041 Registered Nurses (RNs) at an online bachelor's degree program. Descriptive and inferential analysis techniques were performed.

**Results:**

Nurses generally have positive perceptions about online learning (*X *= 3.86; SD = 0.48). A significant difference was seen between nurses who used computers least and those with the highest computer usage [*F *(3, 1033) = 3.040; *P *< .05]. Neither nurses' ages nor lengths of working experience are significantly related to perceptions of online programs (*r *= -.013; *P *> .05 and *r *= -.036; *P *> .05, respectively). Nurses' perceptions are significantly different depending on the settings where they work [*F *(3,989) = 3.193; *P *< .05]. The difference between perceptions of nurses living in urban areas (*X *= 3.82; SD = .51) and those living in rural areas (*X *= 3.88; SD = .47) was not significant [*t *(994) = -1.570, *P *> .05].

**Conclusions:**

We found that nurses regard online learning opportunities as suitable for their working conditions and needs. Nurses should be provided with continued training through online learning alternatives, regardless of age, working experience or area of residence.

## Background

Professionals in developed countries need training to become more qualified and to adapt to changing work conditions. Most of them prefer to get online training [1]. Online learning, which provides learners with a variety of benefits such as convenience, flexibility and opportunities to work collaboratively, has considerable potential for nursing continuing education.

Online nursing continuing education provides Registered Nurses (RNs) with many advantages compared with face-to-face programs. First, it is difficult for nurses to return to school and to become students after a long break. It may also be challenging because of conflicting work schedules, personal responsibilities, and possibly fear of returning to school. More formal learning activities, which provide certifications or degrees, may be more attractive for nurses. However, due to intensive workloads and working shifts, it can be very hard for them to attend face-to-face classes. Advanced online learning technologies are indispensable, particularly for nurses, because of the need for life-long learning and professional development [2,3].

Various online delivery techniques are effective for nursing education [4] due to increased flexibility, accessibility and cost-effectiveness in nursing education [5]. The use of distance learning models in higher education could be a major factor in motivating adult students to return to school [6]. Distance learning can contribute to continuing nursing education and health service quality beyond the fulfillment of professional requirements [7,8].

The number of nurses who participate in online nursing education programs has dramatically increased in recent decades [1-3]. Nurses who wish to join continuous training programs for professional development are glad to have online training options [9]. RNs are able to continue working while pursuing additional online education that will support career advancement [10,11]. Thus, there is a competitive market of online nursing programs due to the increased need for more RNs and flexible online learning [12].

Many studies have shown positive outcomes of online nursing education in terms of achievement, satisfaction, outlook and increased desire for learning. Studies comparing online learning with traditional classroom experiences have shown that academic achievement, socialization, and mentoring opportunities are comparable or improved by using online education [13]. Online nursing training has positive outcomes not only in postgraduate programs such as certification or in-service training but also with bachelor's degree completion or graduate programs [1].

### Perceptions about online learning

There are four factors that contribute to learning: attitude, experience, cognition, and learning style. Attitude is the most important [14]. Positive attitude is favorable for learning because of its influence on learning efficiency, motives, and knowledge application. Generally, the attitudes of students have been very positive and supportive toward online instruction [15].

Students' needs and perceptions should be considered in designing online learning programs. Nurses' perceptions play an important role in program attendance and successful completion. Since the perspectives of online program users are not always consistent with perspectives of course developers or instructors, it is important to understand students' perspectives [16,17]. It is difficult to meet students' learning needs unless one understands what satisfies students in online learning courses [18].

### Theoretical background of study

The Technology Acceptance Model (TAM) provides an important theoretical framework for understanding computer usage and computer acceptance behaviors. The goal of TAM is to provide an explanation of the determinants of acceptance of computer-related applications that depends on two particular perceptions; perceived usefulness and perceived ease of use. This model aids for researchers in identifying acceptance of online programs [19].

Just as the demand for online learning opportunities for RNs has increased, the complexity of providing this type of education has also increased. Nursing faculty are facing the challenges of changing not only their pedagogical styles, but also designing nursing education programs that meet the increased demand for nurses while simultaneously meeting current student needs [20,21].

This study focused on the nurses' perceptions of online continuing education. Dimensions of perceptions and their relationship with personal attributes were also considered. Nurses are required to engage in continuing education, as are other medical professionals. However, it can be difficult for nurses to participate in professional development [22].

Determination of nurses' perceptions of learning programs is important for designing online classes. The number and popularity of online nursing education programs are increasing rapidly in many countries. Understanding these may contribute to designing these programs to maximize success. It may also help to create a training plan for in-service training. The "picture" of nurses' perceptions can be considered as a part of an analysis of the audience. Understanding nurses' perceptions of online learning will provide insight into nursing education challenges, particularly online learning.

This study aimed at revealing nurses' perceptions of online continuing education, and determining these perceptions by area, work setting, frequency of computer usage and age. The research questions that guided this study are:

1. What are the perception levels of nurses of online continuing education?

2. Are there any differences in perception of online continuing education between nurses who use computers more frequently and nurses who use computers less frequently?

3. Is there any relationship between age and perception of online continuing education?

4. Are there any differences between area groups (rural-urban) in terms of perception on online continuing education?

5. Are there any differences in terms of perception of online continuing education between nurses who work in different settings?

## Methods

The survey method was used in this quantitative study to define perception levels and related variables. Data were collected through online instrument from 1041 online student nurses. Descriptive and inferential analysis techniques were performed.

### Sample

The sample was taken from an online nursing bachelor's degree completion program (HELITAM) with an enrollment of 13,200 student RNs. This is the first online nursing program in Turkey. Students are required to have two-year college degrees and be working as nurses in professional settings (e.g., clinics or hospitals) to be admitted to the program.

The study was a convenience sample taken of RNs who were students in the program. The electronic survey was published for a one-week period via Learning Management System. Some 1041 students voluntarily participated in the survey (N = 1041; age range: 25-54 years; 95% female).

### Instrument

Participants were surveyed using a "Student Perceptions Survey of Distance Education" [23]. The instrument asked for demographic information, as well as perceptions of online learning. The questionnaire consisted of 19 items, that measured four factors; perceived matching of conditions and online learning services, perceived usefulness, perceived ease of learning, and characteristics of students.

Perceived usefulness (PU) refers to the degree to which the participants believe that online learning will be effective. This factor focused on perceptions about instructional usefulness.

The second factor, perceived matching of conditions and online learning services (PM), refers to beliefs on how online learning suits learners' needs, lifestyles and situations. This factor is also related to the needs of learners in terms of flexibility and opportunities of online learning. The factor has six items, including "Online learning is suitable for me because of my conflicting work schedule" and "I need the convenience of being able to take courses at my chosen time and place."

The third factor, perceived ease of learning (PE) is adapted from the TAM model, and is related to the ease of taking online courses. It also looks at how students perceive the potential of online programs to support individual learning.

The final item is characteristics of students (CS). This factor is related to individual learning abilities and internal motivation. Items concern "time management," "taking learning responsibility" and "balancing multiple tasks."

The survey's internal reliability was demonstrated by a Cronbach's alpha coefficient of 0.83 [24]. The responses to each perception item were measured on a five-point Likert scale, ranging from "strongly agree" to "strongly disagree."

### Data analysis

In this study, quantitative data were analyzed through descriptive and inferential statistics techniques. Data analysis was performed using SPSS 18. Descriptive analysis was used to measure nurses' perception levels. Correlation analysis was performed to test relationships; ANOVA and independent sample *t*-tests were performed to test differences.

## Results

### Nurses' perceptions level of on online continuing education

The aim of this study was to show nurses' perceptions of online continuing education. Data from the 1041 participants were analyzed descriptively; factors are shown in Table 1. Nurses generally have positive perceptions about online learning (*X *= 3.86; SD = 0.48). The perceived matching of needs and online learning opportunities indicate that nurses have very high perceptions of the personal convenience of online learning (*X *= 4.4; SD = 0.64). However, the nurses' mean score for the efficiency of online learning factor is lower than for other factors (*X *= 3.09; SD = 0.75; *P *< 0.05). The perceived ease of learning factor has a high score (*X *= 3.91; SD = 0.76). The analysis of related aptitude for individual learning shows that nurses' working habits and characteristics are especially suitable for online learning (*X *= 4.13; SD = 0.65).

**Table 1:** Descriptive statistics

Factors	N	Mean	SD
PM	1041	4.41	.64
PU	1041	3.09	.75
PE	1041	3.91	.76
CS	1041	4.13	.65

**Total**	**1041**	**3.86**	**.48**

CS: characteristics of students; PE: perceived ease of learning; PM: perceived matching of conditions and online services; PU: perceived usefulness; SD: standard deviation.

### Effect of computer usage frequency on perceptions of online continuing education

**Figure 1:**
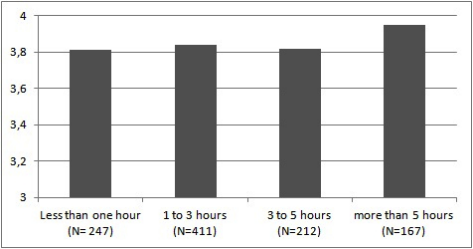
**Perception levels of daily computer usage groups**.

Data about frequency of daily computer usage was collected as ordinal data with 4 options (1: less than one hour; 2: 1-3 hours; 3: 3-5 hours; and 4: more than 5 hours). The perceptions of these groups are shown in Figure 1.

Daily computer usage frequency of groups was compared using an ANOVA test in terms of perceptions; a post hoc test was performed. A significant difference was seen between Groups 1 and 4, but not between any of the other groups [*F *(3, 1033) = 3.040; *P *< 0.05].

### Relationship between age and perceptions of online continuing education

Analysis was performed to determine the correlations of working experience and age of nurses with their perceptions of online continuing education. Age of participants was between 25 and 54 (*X *= 34.5; SD = .12). Neither the nurses' ages nor their lengths of working experience affected their perceptions of online continuing education (*r *= -.013; *P *> .05; and *r *= -.036; *P *> .05, respectively).

### Differences between work area (rural/urban) in terms of perceptions of online continuing education

Of the 1041 responding nurses, 33% worked in rural settings and 67% worked in urban areas. The mean perception of area groups were compared through independent sample *t *tests. The difference between perceptions of urban-based nurses (*X *= 3.82, SD = .51) and rural-based nurses (*X *= 3.88, SD = .47) was not found to be significant [*t *(994) = -1.570, *P *> .05].

### Differences in perceptions of online continuing education among nurses who work in different settings

The settings where nurses worked were classified into five groups; teaching hospitals, general hospitals, hospital clinics and non-hospital clinics. The mean perceptions of work settings groups (Table 2) were compared using ANOVA tests; nurses' perceptions were found to be significantly different according to their work settings [F(3,989) = 3.193, *P *< 0.05]. Post hoc testing results showed that nurses working in general hospitals and hospital clinics have significantly higher perceptions of online learning than nurses from teaching hospitals. Nurses working in hospital clinics have significantly higher perceptions of online learning than nurses from non-hospital clinics.

**Table 2:** Nurses' perceptions by their work settings

	N	PM	PU	PE	CS	Total
		**Mean values**				

General Hospitals	603	4.45	3.11	3.94	4.14	3.89
Hospital Clinic	102	4.51	3.22	3.98	4.17	3.95
Non-Hospital Clinic	121	4.27	3.04	3.97	4.13	3.81
Teaching Hospital	167	4.35	3.0	3.81	4.10	3.79

CS: characteristics of students; PE: perceived ease of learning; PM: perceived matching of conditions and online services; PU: perceived usefulness.

## Discussion

This study aimed to reveal nurses' perceptions of online learning. Results can be interpreted in two aspects; perception level and related variables. Nurses generally have found online learning to be an appropriate educational solution, as many researchers have confirmed [7,8].

When we look at the nurses' perceptions in detail, we found that nurses regard online learning opportunities as suitable for their working conditions and needs. This is related to the flexibility and convenience of online learning. Convenience and flexibility were highly rated among the most important advantages of online learning [24]. Flexibility is also the most satisfying aspect of online learning, as it enables students to study at their own paces and at times convenient to them [25,26].

Results of this study showed that perceived effectiveness of online learning was relatively low, although numerous studies have found online learning is effective in terms of learning outcomes [27,28]. The nurses' relatively low perceptions could be related to lack of online learning experience, since many had never taken online courses previously. Furthermore, some may not be aware of the potentials of online learning technologies.

One result of the study showed that nurses are ready for online learning in terms of learning habits and characteristics. Nurses found it easy to learn online since it provides a personal learning environment. The method of delivery may offer advantages or disadvantages to different students, depending on their characteristics [29]. This is important because certain characteristics such as being "self-directed," or being "independent," are necessary for online learning [30].

The nurses' online learning perceptions are independent of their ages and lengths of work experience. However some studies found that age played an important role in preferring or perceiving online learning [31,32]. This conflict may reflect the fact that participants in this study are working as nurses and most are above thirty years of age.

Results of this study indicate that nurses who use computers more frequently have more positive perceptions of online learning. These results are consistent with research that shows students with more experience with technology rate it more positively [33].

This study found no differences between the perceptions of nurses from rural and urban areas. However another study did find a difference between perceptions based on area of residence [32]. Since all participants are working nurses, there was little difference in income level. Internet availability was similar among respondents. One study did find that nurses from rural area have more desire for online learning. This difference is easily explained by a lack of access to resources such as books, nursing journals, and longer travel distances in rural areas [14]. But in this study all participants had similar access to resources.

There are differences in nurses' perceptions according to their work settings. Nurses working in general hospitals or hospital clinics have significantly higher perceptions of online learning than nurses from teaching hospitals. It can be concluded that nurses working in general hospitals or hospital clinics are more favorably inclined to accept online learning in terms of perceived training needs or working conditions.

## Conclusions

This study revealed the nurses' perceptions of online learning with TAM perspectives and determined the relations of perceptions with some variables. As regarding results of study, the major conclusions are:

• RNs have generally positive perceptions on online learning.

• Online learning fits the needs and working conditions of nurses.

• Online learning suits nearly every age group of RNs.

• Greater computer usage is a predictor of positive perception of online learning.

• Online learning provides flexibility to nurses in both urban and rural areas.

• Nurses working in small-scale health centers may be a priority when designing online nursing programs, as they are the group most inclined to use online education.

The strength of this study is the number of respondents to our survey. Also, results were interpreted from TAM framework perspectives. As this was a quantitative study, its main limitation is that it provided no deeper understanding of respondents' perceptions. The convenience sample size can also be seen as a limitation. The subjects in this study were nurses in Turkey, so the results cannot be generalized to other countries.

Nurses should be provided with online learning alternatives as continuous training regardless of age, working experience or area of residence. Showing good examples to nurses before online education can enhance their perceptions about efficiency of online learning. Nurses' perceptions and learning needs can be deeply investigated as a future study.

## Competing interests

The authors declare that they have no competing interests.

## Authors' contributions

SK was responsible for all aspects of this manuscript.

## Pre-publication history

The pre-publication history for this paper can be accessed here:

http://www.biomedcentral.com/1472-6920/11/86/prepub
